# Study of Preparation and Performance Porous Thermal Insulation Refractory Materials from Aluminum Ash and Red Mud

**DOI:** 10.3390/ma18225253

**Published:** 2025-11-20

**Authors:** Jiayi Zhong, Zichao Li, Weiyuan Li, Hongzhi Yue, Laijun Ma, Haoyu Zhao, Wenjuan Jiao, Yan Wang, Zhiyang Chang

**Affiliations:** 1 Shandong Key Laboratory of Functional-Structural Integrated Ceramics, Discipline and Technology Center for High Temperature Functional Ceramics, College of Materials Science and Engineering, Shandong University of Technology, Zibo 255049, China; 15865619762@163.com (J.Z.); malj4@163.com (L.M.); 19811732833@163.com (H.Z.); jjiaowenjuan@163.com (W.J.); wxiaoy2025@163.com (Y.W.); zychang@sdut.edu.cn (Z.C.); 2 China Building Materials Academy, Beijing 100024, China; lzc990827@163.com; 3 Shandong Industrial Ceramics Research & Design Institute Co., Ltd., Zibo 255086, China; 18678041001@163.com

**Keywords:** aluminum ash, red mud, rheological properties, porous materials, thermal insulation refractories, solid waste resource utilization

## Abstract

The risk of environmental accumulation of aluminum ash and red mud is increasing, emphasizing the demand for high-value utilization. In this study, the conversion of aluminum ash and red mud into porous refractory materials with good thermal insulation performance is successfully demonstrated, demonstrating that both residues can be recovered as a resource and their environmental impact can be reduced in a sustainable manner. The phase composition and microstructure of the waste are evaluated by XRD and SEM/EDS, respectively, while their high-temperature behavior and performance were assessed through visual high-temperature furnace testing. The influence of the aluminum ash-red mud ratio on the rheological behavior of slurries containing surfactants at a constant alkaline pH was highlighted. A slurry composition of 40% red mud and 30% aluminum ash exhibited the lowest shear stress and viscosity values, required to facilitate bubble growth. Building on this formulation, foaming with 2% (mass fraction) H_2_O_2_ at 80 °C and sintering at 1250 °C produces a material with the optimum performance: a compressive strength of 1.03 MPa, a porosity of 58.55%, and thermal conductivity of 0.19 W/(m·K). The material exhibits long-lasting stability at temperatures ≤ 1100 °C. Thus, complementary compositions of aluminum ash and red mud show potential for practical application and value addition in the preparation of porous refractory materials with thermal insulation properties.

## 1. Introduction

Porous insulation refractory materials are a new class of functional materials that are receiving increasing attention for their light weight, low thermal conductivity, high strength, and good stability against thermal shock. They can be divided into alumina-based, silica-based, zirconia-based, and mullite-based materials, among others [1,2,3]. Porous insulation refractory materials are primarily used in construction and aerospace, for example, as infill materials for firewalls in buildings or in aerospace as thermal insulation for components that operate at high temperatures [4,5,6,7]. In addition, they are also widely used as insulation, thermal insulation, and energy-saving materials for industrial thermal equipment [8,9,10]. Currently, the methods commonly used to prepare porous insulation refractory materials include the high-temperature foaming method, 3D printing method, and the low-temperature direct foaming method [11,12,13,14]. The physical properties of porous insulation refractory materials are mainly influenced by their porous structure, and the closed-cell structure is crucial to improving insulation performance [15]. The low-temperature foaming method is straightforward and convenient to carry out, and it offers considerable advantages in pore structure control in the viscous mass or slurry foaming process [16]. The variables affecting the pore structure include factors like the type, performance, and ratios of the raw material, amount of foaming agent, foaming temperature, and sintering temperature.

Secondary aluminum ash (SAA) is a waste byproduct from the aluminum industry, which can have major environmental consequences if not treated in a timely manner. It contains large amounts of alumina, along with some aluminum nitride, magnesium oxide, calcium oxide, and other substances. Its composition is similar to that of bauxite, a raw material for engineering and refractory materials; after sintering at a high temperature, it can form high-temperature-resistant phases, such as corundum, mullite, and magnesialumina spinel [17,18,19]. Therefore, aluminum ash can be used in the production of refractory materials and the ceramic industry. The high-value resource utilization of aluminum ash is important and can promote the sustainability and development of the aluminum industry. However, there is a considerable amount of aluminum nitride in aluminum ash, and this compound has a tendency to react with water to produce ammonia gas, which complicates the resource utilization of aluminum ash [20,21].

Red mud (RM) is the other main solid waste from the aluminum industry. The reddish-brown color of RM is attributed to its hematite content, but it also contains some amounts of alumina, silica, and alkali metal oxides (AMOs), and is composed primarily of ultrafine powder [22,23]. This property suggests its use as a flux to decrease the sintering temperature of ceramics [24,25]. Yet, when used in refractories, AMO can also decrease the refractoriness of the material [26,27]. Some researchers have considered the application of aluminum ash and red mud in the preparation of refractory and ceramic materials. Determining the material composition while considering the characteristics of aluminum dross and red mud poses a challenge to the preparation of porous thermal insulation refractory materials from these two waste products [28,29].

Zhang Yong et al. prepared magnesium alumina spinel refractory materials using aluminum ash as the main raw material and added a certain proportion of magnesium oxide to prepare magnesium alumina spinel refractory materials [30]. At an aluminum ash to magnesium oxide ratio of 1:0.2, high-purity magnesium aluminum spinel was produced by high-temperature sintering, and the compressive strength of the materials was above 40 MPa. Xia et al. utilized red mud and potassium feldspar washing waste (KFW) as raw materials and SiC as a foaming agent to prepare foam ceramics by directly foaming at an elevated temperature (1080–1120 °C). The foam ceramic, prepared with a 1:1 ratio of KFW/RM and 1 wt.% SiC at a sintering temperature of 1100 °C with a sintering time of 60 min, yielded the best overall performance (bulk density, 0.77 g/cm^3^; apparent porosity, 61.89%; average pore size, 0.52 mm; compressive strength, 3.64 MPa). Its excellent porous structure and mechanical properties make it suitable for application as an insulation material or decorative materials in partition walls in buildings [31]. Zong et al. prepared a new type of ceramic with different proportions of red mud and steel slag [32]. The new ceramic with 40% red mud at the optimal sintering temperature of 1140 °C showed the highest bending strength of more than 93 MPa.

In this study, porous insulating refractory materials are produced using aluminum ash and red mud (main materials), calcium aluminate (CA cement/CAC), and bentonite (Be), dosed with sodium polyacrylate (SP) as slurry rheology modifiers, using a wet slurry foaming process (foaming–shaping–firing). The SiO_2_-Al_2_O_3_-CaO ternary phase diagram was used as a guide to modify the high-temperature phases of the material system by adding CA cement (mainly calcium aluminum oxides and corundum), which elevates refractoriness, offsetting the negative effects of red mud and also providing hydration to enhance foam slurry pore stability.

This study systematically examines the effects of slurry properties, foaming process parameters, and sintering schedules on the preparation of high-performance refractory materials from red mud and aluminum ash. The primary innovation of this research is the wet integration foaming–sintering process, which successfully produced a multiphase porous refractory material with good thermal insulation performance. Controlling the slurry pH and surfactant additions, the rheological properties were successfully optimized, establishing an important basis for the production of a stable and modifiable foaming process. Moreover, this work details how the mix ratio, foaming parameters, and sintering schedule interact in a synergistic way to influence a range of parameters which determine the resultant mechanical strength, pore structure, and thermal conductivity. This work not only provides a new and feasible technical pathway for further high-value-added resource utilization of red mud and aluminum ash but also contributes to further promote green development within the refractory industry.

## 2. Materials and Methods

### 2.1. Materials

The aluminum ash was obtained from the Shandong Wei Qiao Aluminum Industry Company in Binzhou, China. The red mud was obtained from the Shandong Branch of Aluminum Industry of China in Zibo, China. The CA cement (CA70 cement) was provided by Zhengzhou Deng Feng Melting Material Co., Ltd. in Zhengzhou, China. The bentonite was provided by Guang Fu Fine Chemical Research Institute in Tianjin, China. The foaming agent was H_2_O_2_, sourced from Fu Chen (Tianjin) Chemical Reagent Co., Ltd. in Tianjin, China. The solvent was deionized water.

Table 1 details the chemical composition of aluminum ash, red mud, CA cement, and bentonite. The major oxide compositions for each of the raw materials are shown as determined by X-ray fluorescence (XRF) analysis. The aluminum ash primarily contains alumina (Al_2_O_3_) and magnesia (MgO), while the red mud primarily contains iron oxide (Fe_2_O_3_), alumina (Al_2_O_3_), and silica (SiO_2_). CA70 cement comprises lime (CaO) and alumina (Al_2_O_3_), while bentonite has a high content of silica (SiO_2_), alumina (Al_2_O_3_), and lime (CaO).

**Figure 1:**
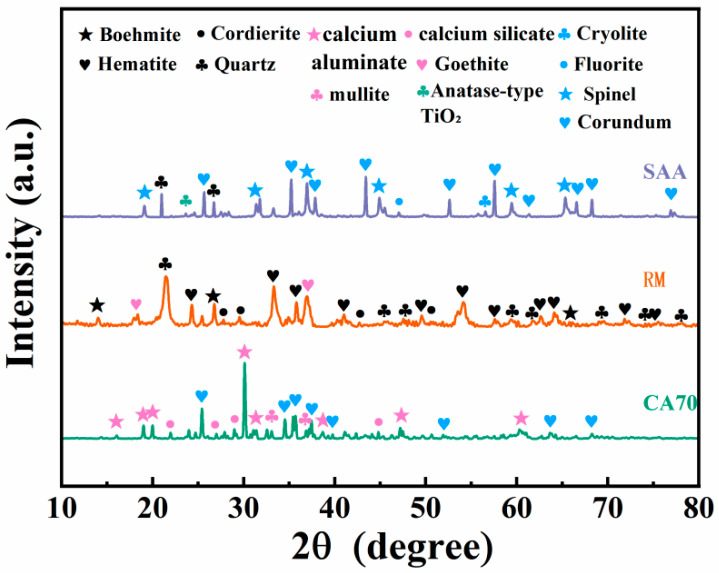
XRD spectra of aluminum ash, red mud and CA cement.

Figure 1 depicts the XRD patterns of the three unprocessed materials, which show different crystalline phase assemblages. The SAA (aluminum ash) has a rather complex composition that largely consists of corundum and metallic aluminum, with fluorine-containing phases such as fluorite and cryolite, as well as lesser quantities of quartz and boehmite. The RM comprises mostly hematite with smaller amounts of quartz, anatase-type TiO_2_, and goethite. The CA70 is primarily composed of calcium aluminate (CaAl_2_O_4_), with some additional phases identified as mullite, calcium silicate (CaSiO_3_), cordierite, and boehmite.

**Figure 2:**
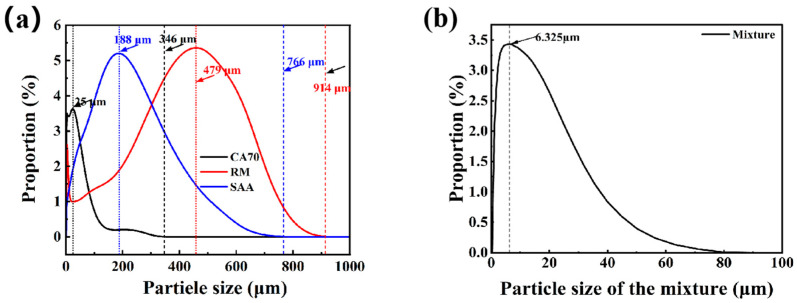
(**a**) Particle size distribution of SAA, CA cement, and red mud. (**b**) Particle size of the mixture.

The particle size distribution curves of aluminum ash, red mud, and CA cement are illustrated in Figure 2. The particle size distribution of red mud is considerably wide, with a range from 0.5 μm to 724 μm, and is concentrated in two areas: 3.80 μm and 478.63 μm. The D50 of red mud is approximately 58 μm. The particle size distribution of aluminum ash is similar to that of red mud, although it is less concentrated, with a primary concentration of 182 μm. The D50 of aluminum ash is approximately 50 μm. The particle size of CA cement is notably smaller than aluminum ash and red mud; the range of particle sizes is approximately 0.5 μm to 100 μm, with a D50 of approximately 20 μm.

### 2.2. Preparation of Porous Ceramics

**Figure 3:**
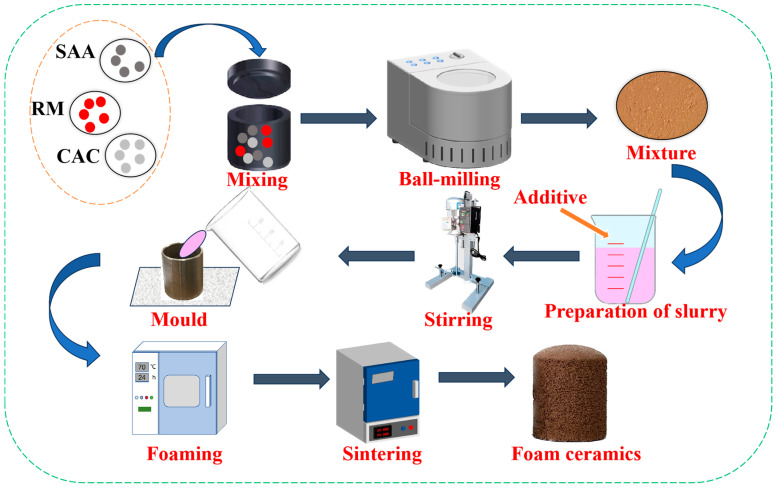
Process flow diagram.

Table 2 shows the composition of raw materials, foaming temperature, and sintering temperature of each sample used in the experiment for preparing porous thermal insulation materials. Figure 3 illustrates the procedure for preparing the porous thermal insulation refractory materials. To ensure more uniform and finer mixed powder, the necessary amounts of aluminum ash, red mud, CA cement, and bentonite are ball-milled for 2 h at a rotation rate of 1500 r/min. The mixed powder is then cast into a beaker and stirred with a glass rod, while adding the required amount of water, sodium polyacrylate, sodium dodecyl sulfate (SDS) and foaming agent, H_2_O_2_. Afterwards, it is stirred for two minutes using a high-speed mixer to ensure that the materials are fully dispersed in the slurry. The slurry is then poured into a metal mold (with a size of φ 50 mm × 50 mm, as shown in Figure 1) and placed in an oven to promote foaming, which takes place at 60~80 °C for 2 h. After foaming, a green body is formed, and prior to removing the slurry from the mold, the slurry is dried for 6 h at 100 °C. The dry green bodies are then sintered for 2 h at the appropriate sintering temperature (based on the sample in question), with a heating rate of 2.5 °C/min. Following the 2 h sintering, the samples are naturally cooled to room temperature in the furnace, completing the preparation of porous thermal insulation materials.

In series A (A1–A6), in which there was a constant foaming temperature of 70 °C, the sintering temperature of 1200 °C, and a foaming agent H_2_O_2_ content of 2 wt.%, the ratio of aluminum ash mass to red mud was varied (from 60:10 to 10:60) to investigate the synergistic use of solid wastes, as well as the role of total aggregate composition in the resulting solid structure of the material.

In series B (B1–B5), while maintaining a fixed ratio of raw materials (aluminum ash/red mud/CA cement/bentonite = 30:40:30:8), foaming temperature (70 °C), and sintering temperature (1200 °C), the content of foaming agent H_2_O_2_ (1–5 wt.%) was varied, to understand the effect of foaming agent dosage on the density and pore structure of the final materials.

In series C (C1–C5), the foaming temperature (60–100 °C) was varied, while keeping the raw material ratio from series B, with a fixed content of foaming agent H_2_O_2_ (2 wt.%) and fixed sintering temperature (1200 °C), to investigate the effect of foaming temperature on the forming behavior of the slurry and the resulting pore uniformity.

In series D (D1–D5), the sintering temperature (1100–1300 °C) was varied, with the same raw material ratio, a fixed content of the foaming agent (2 wt.%), and the optimal foaming temperature (80 °C), to explore the predominant effect of the sintering schedule on the material’s microstructure and the subsequent effect of the microstructure on the mechanical and insulation properties of the final material.

### 2.3. Preparation of Slurry

The DLVO theory provides a quantitative framework for the stability of colloidal particles in dispersed media. Generally, when the zeta potential absolute value on the surface of colloidal particles in a dispersed medium is larger than 30 mV, colloidal particles will be relatively well dispersed through electrostatic repulsion, leading to increased slurry fluidity [33].

This study used a wet slurry foaming–sintering process to develop porous insulation materials, which enable the direct utilization of aluminum ash, following a washing treatment to remove aluminum nitride. To produce slurries that are highly dispersible, the surface zeta potentials of washed and unwashed aluminum ash, red mud, and calcium aluminate cement were systematically studied. The sample dispersion procedure consists of ultrasonic treatment in deionized water to produce a consistent suspension. The pH is adjusted, using a small addition of acid/base solutions, to measure the electrophoretic mobility of charged particles in an electrical field at different pH values. The zeta potential is determined from mobility, and a correlation is established between zeta potential and pH.

**Figure 4:**
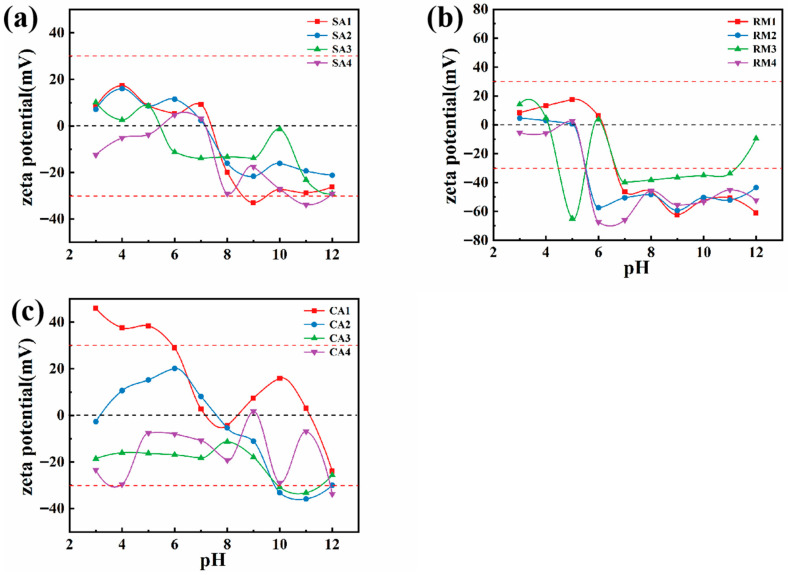
Zeta potential of aluminum ash and red mud in different dispersion environments: (**a**) aluminum ash; (**b**) red mud; and (**c**) CA cement.

Figure 4 depicts the relationship between the particle surface zeta potential and the pH values of the three principal raw materials, using different dispersing medium environments. Figure 4a shows the ζ-pH relationship of aluminum ash. This figure shows that the surface ζ potential of aluminum ash changes significantly after water washing. When the pH exceeds 8, the ζ potential of the washed aluminum ash increases slightly to about −20 mV. However, upon the introduction of sodium dodecyl sulfate and sodium polyacrylate, the ζ potential decreased to approximately −30 mV.

The ζ-pH diagram of red mud is present in Figure 4b, and the ζ-pH diagram of CA cement is displayed in Figure 4c. The figures show that when the pH > 8, the ζ potential of red mud and CA cement was relatively low in deionized water, as well as deionized water that contained the anionic surfactant sodium dodecyl sulfate (SDS) and the non-ionic surfactant sodium polyacrylate (NaPA). The ζ potential of the CA cement is around −30 mV and that of red mud is lower than that of CA cement.

From the analysis in Figure 4, it can inferred that adding a small amount of sodium lauryl sulfate and sodium polyacrylate, while maintaining a pH > 8, provides the proper conditions for preparing a highly dispersible water-based slurry from the mixed powder primarily containing aluminum ash, red mud, and CA cement.

In order to ensure each of the sample slurries had good fluidity when the solid content was ≥50 vol%, the slurry preparation process, based on the zeta potential of each of the raw material particles, was to mix 100 g of powdered material with 8 g of bentonite, 2 g of sodium dodecyl sulfate, 4 g of sodium polyacrylate, and 36 mL of deionized water. The zeta potentials of the three main raw materials of the slurry are shown in Figure 4.

SA1 represents the dispersion system of raw aluminum ash in deionized water, while SA2 to SA4 are systems made from aluminum ash that was soaked in deionized water for 1 h and then allowed to dry naturally before being placed in deionized water containing surfactants. RM1 to RM4 and CA1 to CA4 underwent similar treatments corresponding to the red mud and CA70 cement samples, respectively. In all of the systems, the amount of deionized water, sodium dodecyl sulfate, and sodium polyacrylate were kept constant at 40%, 2%, and 2% of the solid mass, respectively. Each of the prepared samples was allowed to dry naturally, ground to powder, and later used for zeta potential measurements, particle morphology observations, and composition analyses.

The conventional process for preparing a slurry for rheology analysis starts with the preparation of the raw materials. First, the aluminum ash, red mud, CA cement, and bentonite were weighed in the determined ratios and ball-milled at high speeds (1500 r/min, 2 h) to create a dry homogeneous fine mixed powder. Then, the dry powder was mixed with water, sodium polyacrylate (the dispersant), and sodium dodecyl sulfate (the foam stabilizer) sequentially to prepare the slurry. This is then mixed at high shear for two minutes to ensure dispersion of solids and a homogeneous slurry. The mixing sequence, intensity, and duration must all be carefully controlled for repetitive and accurate rheology readings. In the event that a foaming agent such as H_2_O_2_ is required, the mixture is lightly stirred at the end of the mixing of solids and water to avoid generating uncontrolled bubbles, and additional de-aeration by standing may be needed afterwards. The aim is to create slurries of similar composition, stability, and reproducibility that are suitable for rheological study.

### 2.4. Methods

(1) Rheological characterization

The raw materials are mixed according to the formula to obtain a slurry, left to stand at a room temperature of 25 °C for 30 min, and then transferred to the center of the rheometer plate for measurement. The rheological properties are assessed with a rheometer (Haake Mars60, Thermo Fisher Scientific, Waltham, MA, USA) at parallel-plate geometry (1 mm gap). Following a pre-shearing period of time of 300 s, continuous-flow curves are obtained through shear rate sweeps (0.1–100 s^−1^). Dynamic oscillatory tests within the linear viscoelastic region are used to acquire frequency-dependent storage (G′) and loss (G″) moduli (0.1–100 rad/s) at a temperature of 25 °C.

(2) The zeta potential

Zeta potential was measured via electrophoretic light scattering. The raw powder samples (1 mg) were ultrasonically dispersed in deionized water (20 mL) for 1 min, and then allowed to equilibrate for 2–5 min. After the pH adjustment using 0.1 mol/L HCl or NaOH, the zeta potential was recorded at each pH to obtain potential–pH curves.

(3) The compressive strength

The compressive strength of cylindrical samples (φ 50 × 50 mm) is measured using a universal testing machine (CMT4204, Mitter Industrial Systems Co., Ltd., Shanghai, China). The samples are centered on the lower plate and compressed at a speed of 0.5 mm/min while recording the load and displacement until failure occurs. Strength is calculated as σ = F/A, where F is the failure load and A is the cross-sectional area, following GB/T 5072-2023 [34]. For each group, three parallel samples were tested, and the final result is an average of the three parallel sample tests.

(4) The microstructure and pore-size distribution

The microstructure and the pore-size distribution are measured using field emission scanning electron microscopy (Quanta 250, FEI Company, Pantelimon, Romania). Sample cross-sections are gold-sputtered and imaged with 15 kV/10 mm working distance. Pore-size distributions can be determined from secondary electron imaging by Nano Measure 1.2 software, using grayscale thresholding. By setting a grayscale threshold, the image is binarized into black and white (black representing voids and white representing the matrix), and then the geometric parameters of each black region (such as area and perimeter) are measured, and their distributions are analyzed.

(5) The porosity

Apparent porosity was assessed using the Archimedes method. Saturated samples (3 h boiling in distilled water) were weighed for their dry weight (M1), saturated weight (M2), and suspended weight (M3). The porosity is expressed as [(M2 − M1)/(M2 − M3)] × 100%, in line with GB/T 2997-2015 [35]. The reported results for each measurement are the average of three parallel samples for each group.

(6) X-ray diffraction (XRD) analysis

The phase composition was assessed using an X-ray diffractometer (Bruker D8 Advance, Bruker Corporation, Billerica, MA, USA). Powder samples were packed flat into glass holders and then examined for phase characterization using copper Kα (λ = 1.5406 A), scanned at a rate of 4°/min (step size 0.02°). The phases were identified by comparing the resulting patterns to the ICDD using Jade software 6.5.

(7) X-ray fluorescence (XRF)

The chemical composition of the raw materials was measured using X-ray fluorescence spectroscopy (ZSX100e, Rigaku Corporation, Tokyo, Japan). Specifically, powder samples were pressed into flattened pellets, placed in the sample chamber, and subsequently X-ray exposed to stimulate element-specific fluorescence. The X-ray fluorescence detector evaluated the characteristic peak intensity of elements present in the sample to estimate the oxide composition.

(8) Particle size distribution

The particle size distribution of the raw materials was determined by using a laser particle size analyzer (Malvern Mastersizer 2000, Malvern Panalytical, Malvern, UK) via the wet method. Specifically, the powdered sample was inserted into a circulation disperser, where it was ultrasonically dispersed in deionized water. Subsequently, the light scattering intensity of the materials was measured using laser diffraction, whereas the particle size distribution was measured based on Mie theory, expressed as a volume distribution.

(9) The refractory properties

The refractory properties were assessed in a systematic manner using three measures: refractoriness, reheating linear change, and strength loss rate after heating. All measures were completed according to the national Chinese standards: for example, refractoriness was assessed according to GB/T 7322-2017 using a pyrometric cone method of assessing temperature at the material’s softening endpoint [36]; reheating linear change was measured according to GB/T 5988:2022 by dimension changes, before and after 3 h of soaking at a set temperature (e.g., 1250 °C) [37]; the strength loss rate was measured according to GB/T 5072-2023 to examine the difference in cold crushing strength between sets of identical samples before and after reheating, which indicates high-temperature durability [34].

## 3. Results

### 3.1. Rheological Analysis

**Figure 5:**
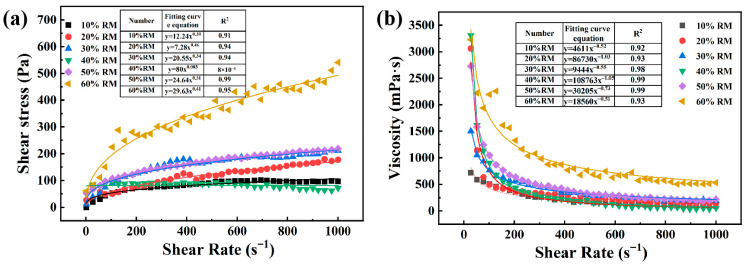
Relationship between shear stress and viscosity of slurry and shear rate for different aluminum ash/red mud ratios: (**a**) shear stress; (**b**) viscosity.

The rheological behavior of the slurries containing different red mud/aluminum ash combinations is illustrated in Figure 5. The flow curves were modeled using the Bingham model (τ = τ_0_ + μ$\dot{\gamma }$), where τ_0_ is the yield stress and μ is the plastic viscosity. All slurries exhibited normal shear-thinning conditions as the shear stress increased, and the viscosity decreased with the rise in shear rate. At red mud concentrations < 50 wt.%, the slurries exhibited yield stresses < 180 Pa and plastic viscosities < 950 mPa·s, demonstrating good flowability [38]. In fact, the slurry formulation with 40% red mud exhibited the smallest Bingham parameters (τ_0_ = 152 Pa, μ = 882 mPa·s), where low-yield stress creates favorable conditions for the nucleation of bubbles and low viscosity reduces the resistance to bubble growth. This enhances the buoyancy-induced rearrangement of bubbles and creates a more homogenous pore structure. On the other hand, red mud concentrations > 60 wt.% led to a dramatic increase in the Bingham parameters (τ_0_ > 380 Pa, μ > 1450 mPa·s). On average, shear stresses were greater than 400 Pa with viscosities > 1500 mPa·s at a low shear rate (≤200 s^−1^). With increased yield stress and viscosity, the activity of the bubbles is compromised through limited nucleation, limited growth, and compromised migration. This results in non-homogenous bubble distribution and macro-structure coarsening. Highly viscous slurries even limit fill amplitude and lead to air entrapment and structural defects, further decreasing the macro-homogeneity and process stability of the slurry.

### 3.2. Compressive Strength Analysis

**Figure 6:**
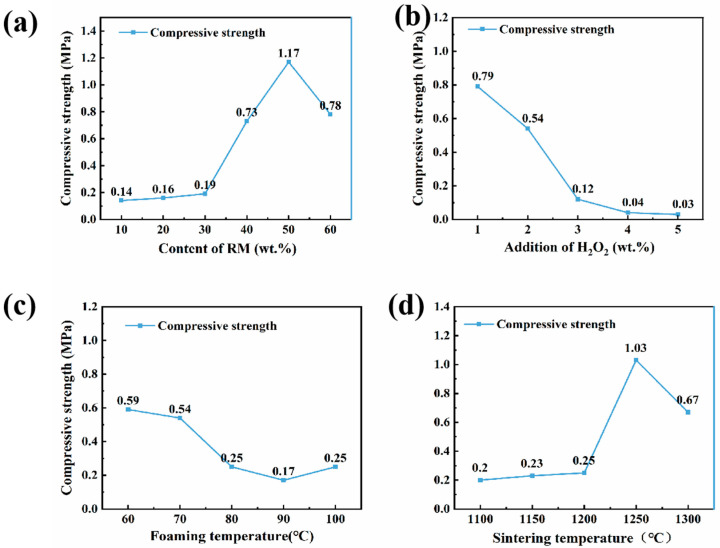
The effect of different conditions on the compressive strength of porous insulation materials: (**a**) different red mud contents; (**b**) different concentrations of H_2_O_2_; (**c**) different foaming temperatures; and (**d**) different sintering temperatures.

The influence of various factors on the compressive strength of the porous insulation materials is illustrated in Figure 6 (tested by following GB/T 5072-2023). Figure 6a shows the effect of the red mud/aluminum ash ratio, and the tested samples correspond to A1–A6 in Table 2. The results show that compressive strength increased to a peak and then reduced, and it exceeded 50% red mud addition. This occurred because, while aluminum ash was the dominant material, the high alumina content necessitated a higher sintering temperature. In this case, the correct sintering state was not achieved at 1200 °C. When there was a modest amount of red mud, it increased the silica/alumina ratio and increased liquid phase formation to promote sintering, resulting in improved strength. If there was a higher addition of red mud, the alumina content, for example, would have unnaturally reduced and therefore caused excessive liquid phase and reduced crystalline phases at 1200 °C, which would have decreased the mechanical properties. Figure 6b illustrates the effect of H_2_O_2_ foaming agent dosage, and the tested samples correspond to B1–B5 in Table 2. Compressive strength decreased from 0.79 MPa to 0.03 MPa as the foaming agent concentration increased from 1% to 5%. Since the foaming agent dosage was 2%, the strength was 0.54 MPa, but as dosage increased to 3%, it fell sharply to 0.12 MPa. This was likely due to excessive gas generated with higher foaming agent concentrations, resulting in pore coalescence or rupture. It was therefore noted that the optimal dosage of the H_2_O_2_ foaming agent was 2%. Figure 6c illustrates the effect of the foaming temperature, with tested samples corresponding to C1–C5 in Table 2. The compressive strength result shows that strength decreased when the foaming temperature changed from 60 °C to 100 °C. This could be attributed to higher foaming temperatures increasing the decomposition rate of H_2_O_2_, thereby leading to concentrated gas release and encouraging uneven pore coalescence or rupture, and therefore decreasing strength. If foaming temperatures were below 60 °C, the extended foaming duration likely caused the CA cement to solidify prematurely, thus limiting pore formation. The suggested ideal foaming process temperature was between 60 °C and 80 °C. Figure 6d shows the effect of the sintering temperature, with samples corresponding to D1–D5 in Table 2. Compressive strength led to the conclusion that there was an increase and then decrease in compressive strength, rising from 1100 °C to 1300 °C with a red mud content of 40%. The results also suggested that the peak occurred at 1250 °C. Until 1250 °C, the intensity of liquid phase formation promoted sintering density, which would improve strength. Once the temperature was at 1250 °C, the intensity of the liquid phase would be too high, negatively affecting its mechanical properties.

### 3.3. Porosity and Thermal Conductivity

**Figure 7:**
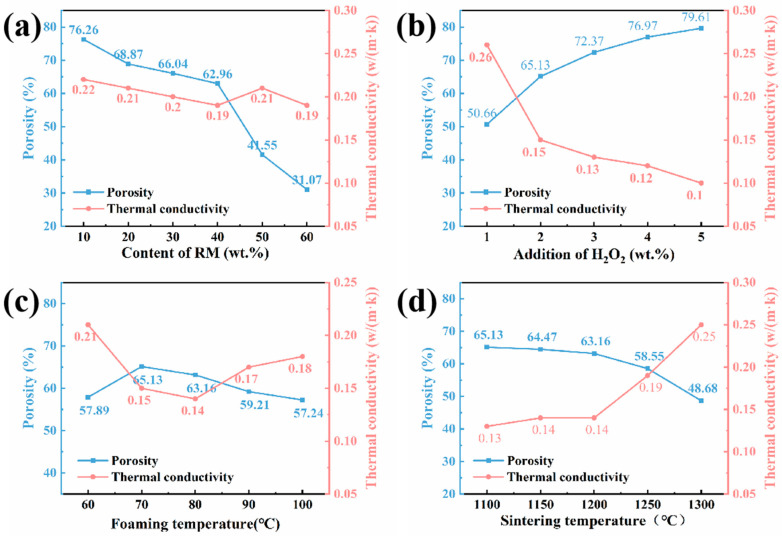
The influence of pore-size distribution and thermal conductivity of porous insulation materials under different conditions: (**a**) different red mud contents; (**b**) different concentrations of H_2_O_2_; (**c**) different foaming temperatures; and (**d**) different sintering temperatures.

Figure 7 illustrates the impact of the pore-size distribution and thermal conductivity of porous insulation materials under different conditions. As seen in Figure 7a, the porosity decreases from 76.26% to 31.07%. As the amount of red mud increases, there is no significant difference in the thermal conductivity of the sample. In Figure 7b, it can be seen that as the amount of foaming agent added increases, the porosity increases from 50.66% to 79.61%. At a content of 2 wt.% H_2_O_2_, the compressive strength is highest under good thermal conductivity conditions (0.15 W/(m·k)). In Figure 7c, as the foaming temperature increases, the pore size first increases and then decreases, reaching a maximum of 70 °C. As the foaming temperature increases, thermal conductivity first decreases and then increases. The thermal conductivity is lowest, with a value of 0.14 W/(m·k) at 80 °C, and a porosity of 63.16%. In Figure 7d, as the sintering reaches its maximum at 1250 °C, the porosity decreases, and the thermal conductivity gradually increases. Strength reaches its maximum at 1250 °C at 1.03 MPa and a thermal conductivity of 0.19 W/(m·k). In conclusion, to use the porous insulation material, it must have high compressive strength and maintain good thermal conductivity properties when applied to refractory insulation boards in kilns. Therefore, samples at 1250 °C are considered to improve the properties of the porous insulation material.

### 3.4. XRD Analysis

#### 3.4.1. XRD Spectra of Porous Insulation Materials Prepared with Different Aluminum Ash/Red Mud Ratios

**Figure 8:**
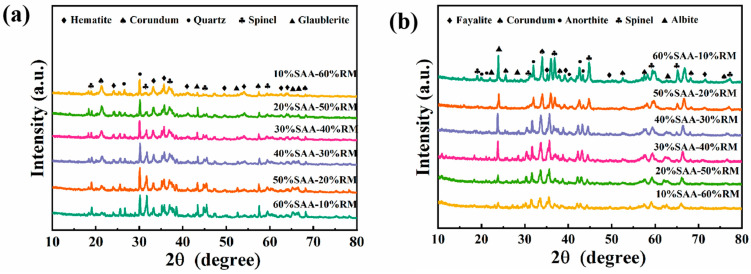
XRD spectra of porous insulation materials prepared with different aluminum ash/red mud ratios (**a**) before sintering and (**b**) after sintering.

In Figure 8, the phase changes in different samples, based on varying SAA-RM ratios, before and after sintering are presented. As the red mud increases in content, the alumina phase (corundum phase) and aluminum nitride decrease in the starting material. The increase in Si/Al ratio facilitates the liquid phase during sintering, which affects the performance of porous insulation materials. The Si/Al ratio discussed in this work is defined as the molar ratio of SiO_2_ to Al_2_O_3_ in the overall oxide composition of the blended raw materials (aluminum ash, red mud, and CA cement)—this was determined via XRF elemental analysis. Figure 8 displays the phase composition of the porous insulation materials made with different aluminum ash/red mud ratios after sintering. As the quantity of aluminum ash decreases, the amount of magnesium aluminum spinel and calcium magnesium compounds decreases, while the amount of iron olivine increases and then decreases with increased red mud content. This may be attributed to the incorporation of a large amount of iron from the red mud, which probably combines with another material to form new phases when the red mud content is very high.

#### 3.4.2. XRD Spectra of Porous Insulation Materials Prepared at Different Sintering Temperatures

**Figure 9:**
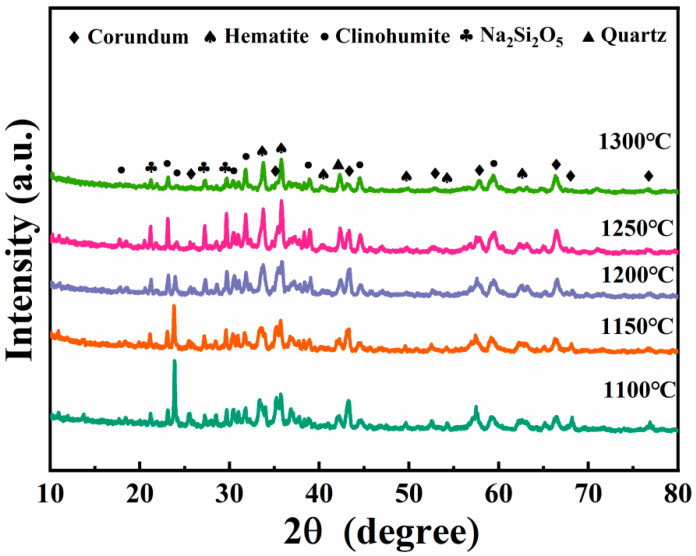
XRD spectra of porous insulation materials prepared at different sintering temperatures (D1–D5).

Figure 9 illustrates the XRD spectra of porous thermal insulations made at sintering temperatures of 1100 °C, 1200 °C, and 1250 °C. At the 1100 °C sintering temperature, porous insulation materials are predominantly composed of corundum, magnesium aluminum iron oxide, garnet, aluminum silicon nitride oxide, magnesium aluminum spinel, and iron fluoride. The corundum, magnesium aluminum spinel, and iron fluoride contents decline with an increased sintering temperature. The highest levels of magnesium silicon nitride, iron olivine, sodium silicate, and aluminum sodium silicate phases were identified at the sintering temperature of 1250 °C.

### 3.5. SEM and EDS Analysis

#### 3.5.1. SEM-EDS and Pore-Size Distribution of Porous Insulation Materials Prepared with Different Aluminum Ash/Red Mud Ratios

**Figure 10:**
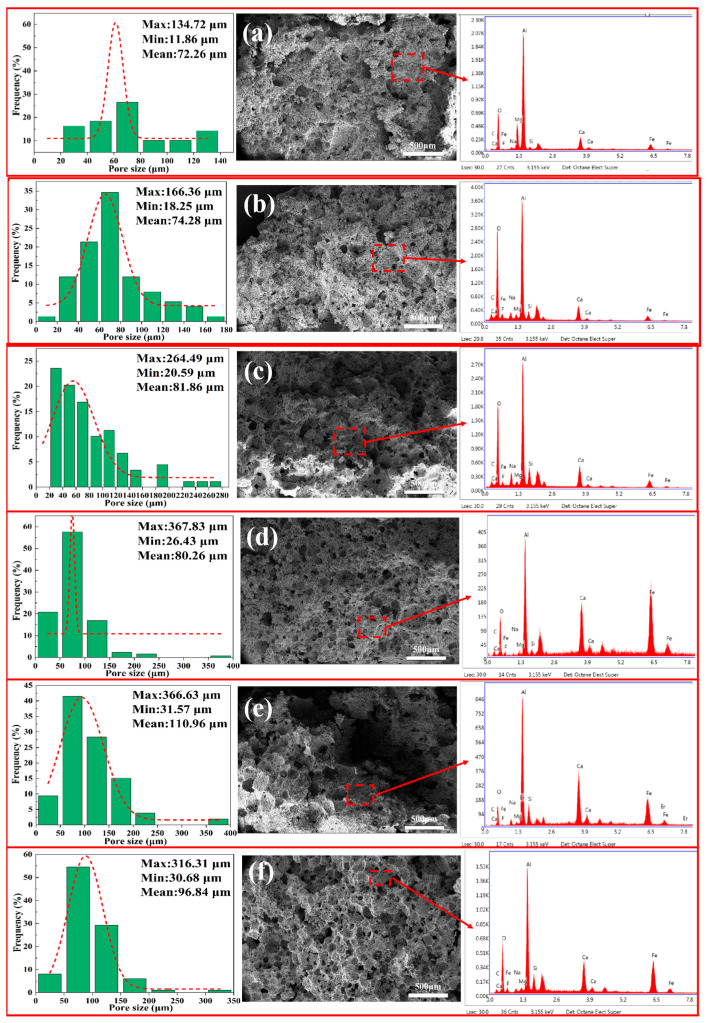
SEM-EDS and pore-size distribution of porous insulation materials prepared with different aluminum ash/red mud ratios: (**a**) 60% SAA-10% RM; (**b**) 50% SAA-20% RM; (**c**) 40% SAA-30% RM; (**d**) 30% SAA-40% RM; (**e**) 20% SAA-50% RM; and (**f**) 10% SAA-60% RM.

As presented in Figure 10, the quantitative statistical results indicate that a trend can be observed in the pore structure of samples with varying formulations. Specifically, as the red mud content increased, the average pore size increased from 72.26 μm in Sample A to 96.84 μm in Sample F, while the maximum pore size increased from 134.72 μm to 316.31 μm. Out of all the samples, Sample F (40% red mud) showed ideal pore-size distribution characteristics, as it had a high concentration and unimodal distribution curve, and its SEM images showed consistent pore morphology and intact pore walls. These results further support the conclusion drawn from prior rheological tests, which indicated that this formulation was most beneficial to stable bubble growth [39,40].

#### 3.5.2. SEM and Pore-Size Distribution of Porous Insulation Materials Prepared with Different Amounts of H_2_O_2_ Addition

**Figure 11:**
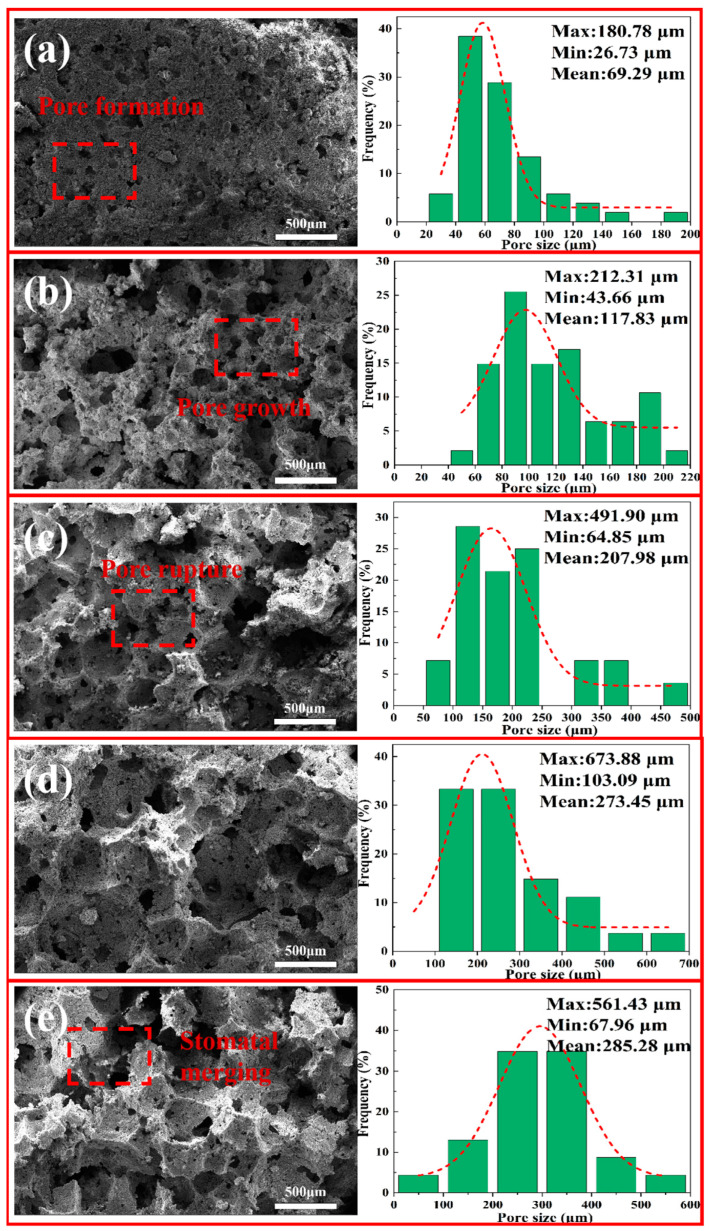
SEM and pore-size distribution of porous insulation materials prepared with different amounts of H_2_O_2_ addition: (**a**) 1% H_2_O_2_; (**b**) 2% H_2_O_2_; (**c**) 3% H_2_O_2_; (**d**) 4% H_2_O_2_; and (**e**) 5% H_2_O_2_.

Figure 11 shows SEM microstructure images and pore-size distribution diagrams of the samples (B1–B5) with different amounts of H_2_O_2_ foaming agent. As can be seen, the samples have a distinctly different microstructure. The sample with 1% H_2_O_2_ has a lower number of pores and smaller pore sizes, with an average size of approximately 69 μm. As the H_2_O_2_ foaming agent amount increases to 5%, the pore number significantly increases, and the pore size also increases significantly, with an average of around 285 μm. After the amount of H_2_O_2_ foaming agent exceeds 3%, merged and ruptured pores are visible, especially with 5% H_2_O_2_ foaming agent. Overall, Figure 11 depicts that after the foaming agent has created the pores in the materials, the pore undergoes a process of generation, growth, rupture, and merging, which also shows that the amount of foaming agent has a significant effect on the material’s microstructure [41,42].

#### 3.5.3. SEM and Pore-Size Distribution of Porous Insulation Materials Prepared at Different Foaming Temperatures

**Figure 12:**
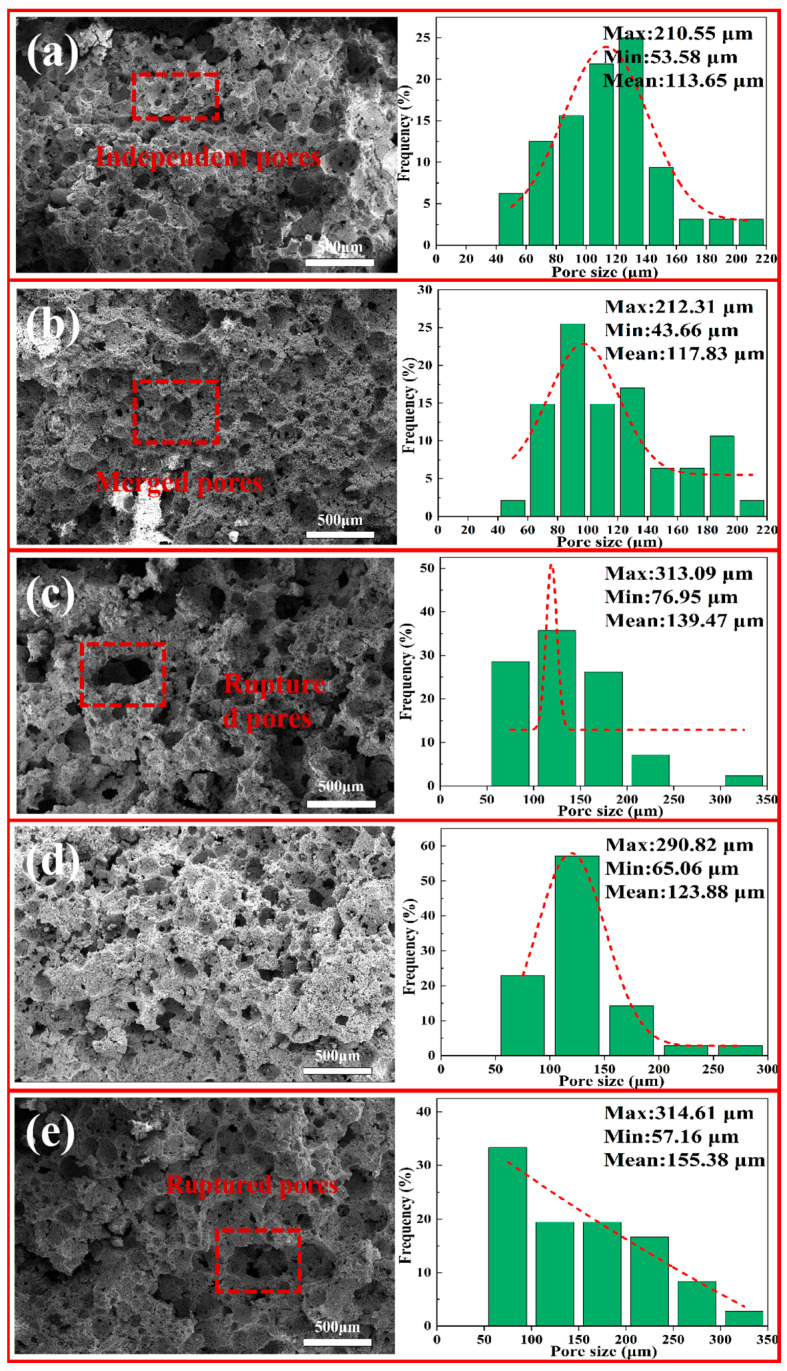
SEM and pore-size distribution of porous insulation materials prepared at different foaming temperatures: (**a**) 60 °C; (**b**) 70 °C; (**c**) 80 °C; (**d**) 90 °C; and (**e**) 100 °C.

Figure 12 depicts the SEM microstructure images, along with the pore-size distribution of samples (C1–C5) sintered at 1200 °C, following foaming at different temperatures. As the foaming temperature rises, the size of the pores tends to gradually increase. In the sample foamed at 60 °C, there are still a few regions that are free of pores and where pore size is relatively small. This is mainly due to insufficient decomposition of the foaming agent at this temperature, which leads to inadequate pore generation and insufficient growth. For the samples foamed at 70 °C and 80 °C, the foaming agent underwent much better decomposition, increasing both the number of pores and their size, and also improving uniformity. As the foaming temperature reaches 90 °C, the number of broken and merged pores begin to increase, the pore size enlarges, and uniformity decreases. This trend becomes more pronounced at a foaming temperature of 100 °C.

#### 3.5.4. SEM and Pore-Size Distribution of Porous Insulation Materials Prepared at Different Sintering Temperatures

**Figure 13:**
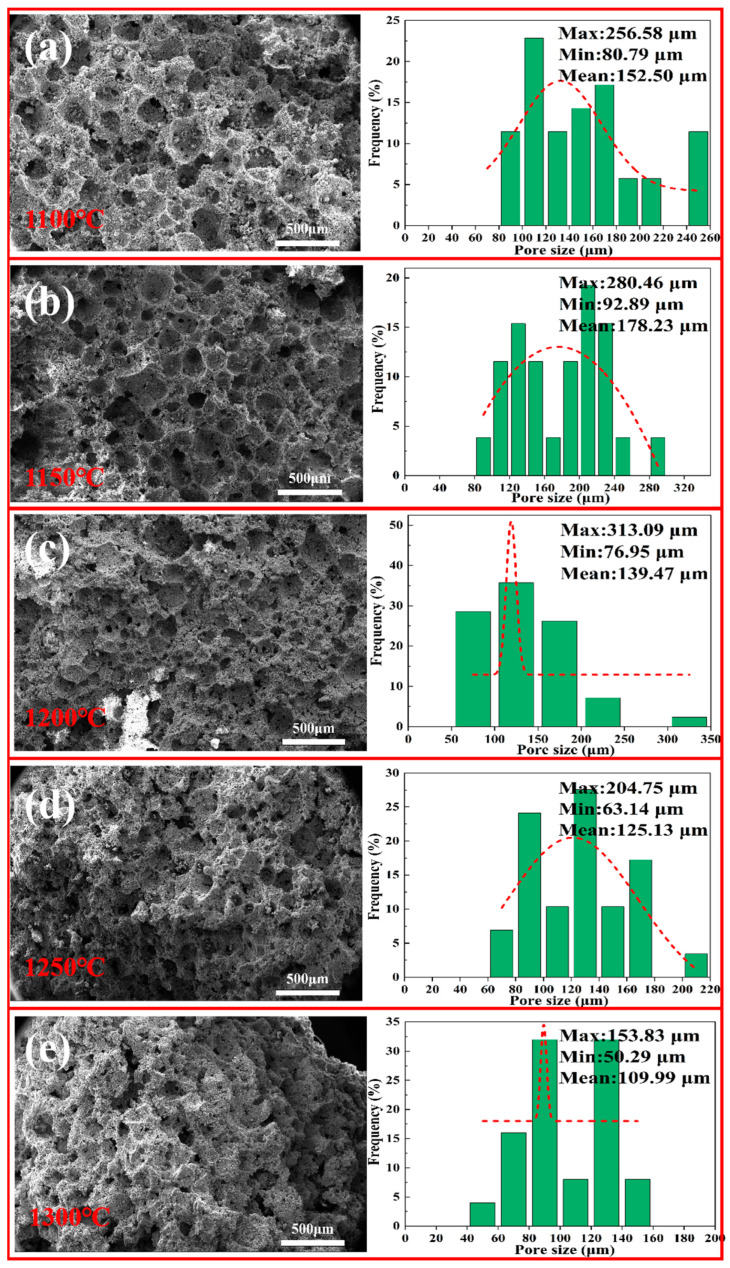
SEM and pore size distribution of porous insulation materials prepared at different sintering temperatures: (**a**) 1100 °C; (**b**) 1150 °C; (**c**) 1200 °C; (**d**) 1250 °C; (**e**) 1300 °C.

Figure 13 shows SEM microstructure images and pore-size distribution curves of samples (D1–D5) processed at different sintering temperatures. Observations from the figure show that the pore size of the porous material decreases as the sintering temperature increases. As the sintering temperature rises from 1100 to 1300, the pore size of the samples decreases from 178 μm to 109 μm, which is consistent with earlier results regarding the effect of sintering temperature on porosity. With the increase in temperature, the fraction of small pores in the 30–40 μm range drastically decreases, which suggests that with the increasing liquid phase in each sample, the limited pores are eventually filled, causing large pores to decrease in size and limited small pores to eventually disappear, decreasing overall porosity.

### 3.6. Fire Resistance Performance

#### 3.6.1. Refractoriness

**Figure 14:**
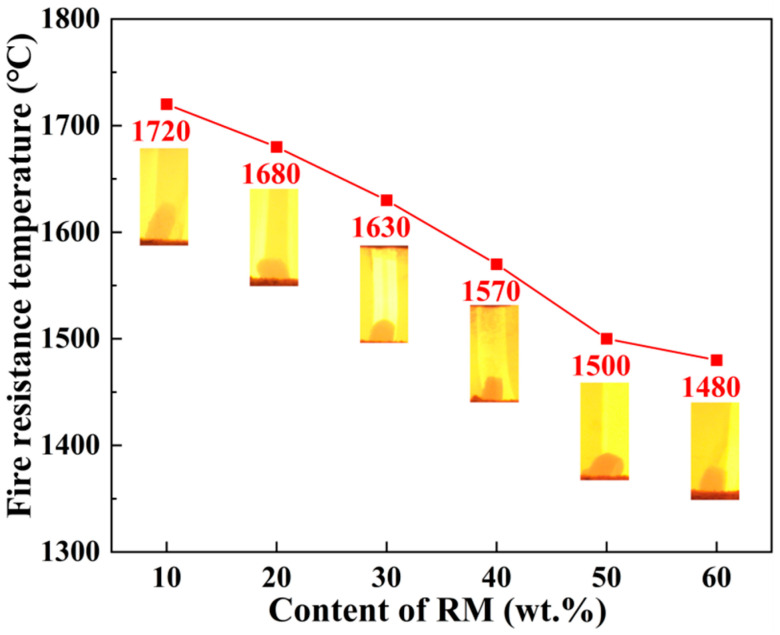
Fire resistance of porous insulation materials prepared with different aluminum ash/red mud ratios.

**Figure 15:**
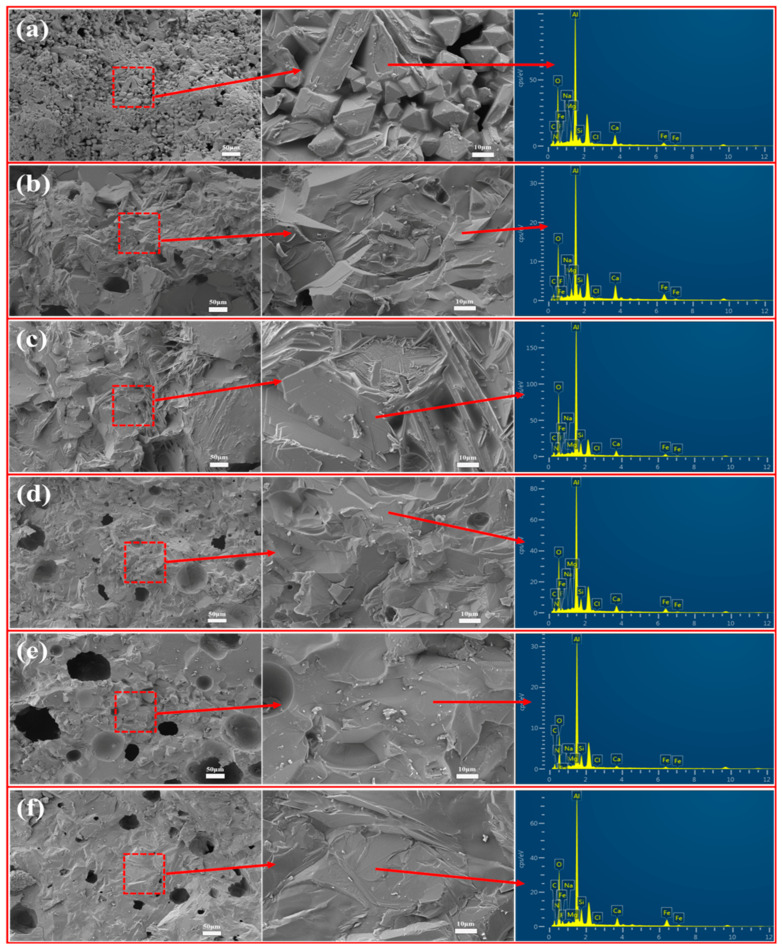
SEM-EDS analysis of specimens with different aluminum ash/red mud ratios after refractoriness testing (**a**) 60% SAA-10% RM; (**b**) 50% SAA-20% RM; (**c**) 40% SAA-30% RM; (**d**) 30% SAA-40% RM; (**e**) 20% SAA-50% RM; and (**f**) 10% SAA-60% RM.

This research highlights refractoriness, linear change after reburning, and strength loss after reburning to characterize the refractory properties of the insulation materials prepared from aluminum ash and red mud. Figure 14 shows the effect of aluminum ash/red mud content on the refractoriness of porous insulating materials. The tested samples are represented as A1–A6 in Table 2. The refractoriness values of aluminum ash/red mud are shown in the figure, with photographs of the sample melting morphology taken at the temperature presented below each corresponding refractoriness value. The figure shows that adding red mud significantly lowers the obtained refractoriness values in the range of 1720–1480 °C. Sample A1 maintained an increased residual sample height compared to samples 2–4, which melted and collapsed, indicating that as more red mud was added to the samples, less Al_2_O_3_ content was preserved in the sample, allowing that amount of high-temperature liquid phase to build. Al_2_O_3_ content correlates directly with the increased refractoriness of the material. However, when the aluminum ash content is 30 wt.% and red mud content is 40 wt.%, the refractoriness value of the porous insulating material reaches the 1570 °C range, which approaches the basic requirement for refractoriness of medium-temperature refractory materials. For medium-temperature refractory materials, the refractoriness should not be less than 1580 °C. However, it must be noted that this applies for any use of the red mud–aluminum ash refractory combination and should be taken into account for end-product-use temperature when producing red mud–aluminum ash refractory materials. To investigate any microstructural changes in aluminum ash/red mud insulating refractory materials subjected to high-temperature deformation, SEM-EDS observations were carried out on the residual parts after testing the refractoriness. The residual parts from the refractory materials are shown in Figure 15 with their SEM image and EDS spectrum. The images show that after refractoriness testing, a majority of the pores in the samples are filled with a liquid phase formed at high temperature, with a notable decrease in porosity. The EDS results indicated that the liquid phases are predominantly composed of an aluminosilicate phase, containing sodium, calcium, and oxygen. The images indicate that more red mud in the sample led to more liquid phases. Furthermore, in sample A1, with only 10% red mud content, there was very little liquid phase presence after testing the refractoriness at 1720 °C, possibly due to the presence of larger granules and needle-like crystals, which prevented the melting/collapse of the structure.

#### 3.6.2. Durability

The changes in performance of the refractory materials after secondary sintering are very important for evaluating their durability. These changes can be expressed through changes in strength and dimensional linearity after secondary sintering. Table 3 shows the strength loss rate and dimensional linearity change rate of porous material samples with 30% aluminum ash and 40% red mud, after being sintered at 1200 °C, followed by secondary heat treatment at 1100 °C for 2 h. The calculation formulas are shown below.

(1) $$\mathrm{Strength}\;\mathrm{loss}\;\mathrm{rate}:\;\Delta F=(F_{1}-F_{2})/F_{2}$$

(2) $$\mathrm{High}\;\mathrm{loss}\;\mathrm{rate}:\;\Delta H=(H_{1}-H_{2})/H_{1}$$

(3) $$\mathrm{Diameter}\;\mathrm{loss}\;\mathrm{rate}:\;\Delta D=(D_{1}-D_{2})/D_{1}$$

*F*_1_: Sample strength before secondary sintering/MPa; *F*_2_: sample strength after secondary sintering; H_1_: sample height before secondary sintering/mm; H_2_: sample height after secondary sintering/mm; D_1_: sample diameter before secondary sintering/mm; D_2_: sample diameter after secondary sintering/mm.

According to the parameters shown in Table 4, the strength loss rate and the volume loss rate of the sample after re-sintering at 1100 °C are less than 0.2%, indicating that the sample can be assessed as a low-to-medium-term refractory material used for prolonged service life.

## 4. Conclusions

Aluminum ash and red mud are common solid wastes produced in the aluminum industry. Their utilization is of great significance for the sustainable development of this industry. In this study, we investigated a wet process featuring foaming and sintering to produce porous insulation refractory materials from aluminum ash and red mud. We studied the effects of the rheological properties of water-based slurries of mixed aluminum ash and red mud powder. Moreover, the properties of the porous insulation materials were studied at different aluminum ash/red mud ratios, sintering temperatures, foaming agent dosages, foaming temperatures, etc. By controlling the pH value and adding surfactants, the rheological properties of the slurries of aluminum ash and red mud mixed powder were optimized to a state suitable for foaming. The slurry with less than 50 wt.% red mud had an average shear stress of less than 200 Pa and an average viscosity of less than 1000 mPa·s, indicating good fluidity. The mixed powder slurry containing red mud at 40% showed the lowest shear stress and viscosity, which was favorable for pore formation and growth in foams. When the red mud content exceeds 60 wt.%, the average shear stress exceeds 400 Pa, and in the low-shear-rate region (<200 s^−1^), the slurry viscosity is higher than 1500 mPa·s. This indicates a great decline in the slurry’s rheological properties, which does not favor stability. Important factors that influence the properties of porous insulation materials are the aluminum ash and red mud ratio, sintering temperature, foaming agent amount, and foaming temperature. At aluminum ash and red mud amounts of 30 wt.% and 40 wt.%, respectively, with a H_2_O_2_ foaming agent amount of 2 wt.%, a foaming temperature of 80 °C, and a sintering temperature of 1250 °C, a relatively good porous insulation material is prepared, with a compressive strength of 1.03 MPa, porosity of 58.55%, and thermal conductivity of 0.19 W/(m·K).

The refractory properties and durability of porous insulation materials at a high temperature are represented by refractoriness, linear change after reburning, and strength loss after reburning. Upon increasing the red mud amount, the refractoriness of samples declines significantly from 1720 °C to 1480 °C. The sample with an aluminum ash content of 30 wt.% and red mud content of 40 wt.% has a refractoriness of 1570 °C, which is similar to the performance of medium-temperature refractory materials (minimum refractoriness of 1580 °C). Also, the strength loss rate and linear change rate of the sample produced with this raw material ratio are lower than 0.2% following reburning at 1100 °C for 2 h. This implies that an aluminum ash–red mud porous refractory material produced with this raw material ratio can be used reliably in long-term applications as a medium- or low-temperature refractory material.

Based on the above findings, the next phase of research will include the introduction of an industrial solid waste (i.e., steel slag, fly ash) or functional powders (i.e., silicon carbide, titanium dioxide) to study the synergistic regulation of the thermal conductivity, mechanical strength, and high-temperature resistance of the material. Further exploration of the dual value of “collaborative utilization of solid waste”, with regard to environmental protection and economic benefits, should also be undertaken to further enhance the cost-effectiveness of this material.

## Figures and Tables

**Table 1:** Chemical composition of raw materials/wt.%.

Component	Al_2_O_3_	CaO	MgO	Na_2_O	SiO_2_	Fe_2_O_3_	K_2_O	Cl
Red mud	16.50	1.71	0.28	7.09	12.00	48.00	0.18	0.06
Aluminum ash	63.6	0.828	9.23	8.78	2.64	0.446	2.31	6.62
CA cement	68.7	29.2	0.173	0.32	1.12	0.276	0.0254	0.0078
Bentonite	12.67	7.77	1.72	0.24	73.98	1.25	2.02	0.02

**Table 2:** Raw material composition (wt.%), foaming temperature (°C), and sintering temperature (°C) used in the preparation of porous insulation materials.

Sample	SAA	RM	CAC	SDS	Be	SP	H_2_O_2_	Foaming Temperature	Sintering Temperature
A1	60	10	30	+2	+8	+4	2	70	1200
A2	50	20	30	+2	+8	+4	2	70	1200
A3	40	30	30	+2	+8	+4	2	70	1200
A4	30	40	30	+2	+8	+4	2	70	1200
A5	20	50	30	+2	+8	+4	2	70	1200
A6	10	60	30	+2	+8	+4	2	70	1200
B1	30	40	30	+2	+8	+4	1	70	1200
B2	30	40	30	+2	+8	+4	2	70	1200
B3	30	40	30	+2	+8	+4	3	70	1200
B4	30	40	30	+2	+8	+4	4	70	1200
B5	30	40	30	+2	+8	+4	5	70	1200
C1	30	40	30	+2	+8	+4	2	60	1200
C2	30	40	30	+2	+8	+4	2	70	1200
C3	30	40	30	+2	+8	+4	2	80	1200
C4	30	40	30	+2	+8	+4	2	90	1200
C5	30	40	30	+2	+8	+4	2	100	1200
D1	30	40	30	+2	+8	+4	2	80	1100
D2	30	40	30	+2	+8	+4	2	80	1150
D3	30	40	30	+2	+8	+4	2	80	1200
D4	30	40	30	+2	+8	+4	2	80	1250
D5	30	40	30	+2	+8	+4	2	80	1300

**Table 3:** Formulas for adding different additives to aluminum ash, red mud, and CA cement in sequence.

	SAA	RM	CA	H_2_O	SDS	SP
SA1	√	-	-	-	-	-
SA2	√	-	-	√	-	-
SA3	√	-	-	√	√	-
SA4	√	-	-	√	√	√
RM1	-	√	-	-	-	-
RM2	-	√	-	√	-	-
RM3	-	√	-	√	√	-
RM4	-	√	-	√	√	√
CA1	-	-	√	-	-	-
CA2	-	-	√	√	-	-
CA3	-	-	√	√	√	-
CA4	-	-	√	√	√	√

**Table 4:** Changes in compressive strength and diameter of porous insulation materials before and after secondary sintering.

Items	Sintering Temperature (°C)	Early Stage	Later Stage	Loss Rate (%)
Compressive strength (MPa)	1000	1.038	1.037	0.096
	1100	1.036	1.034	0.193
Diameter (mm)	1000	44.35	44.32	0.068
	1100	43.91	43.83	0.182
Height (mm)	1000	36.83	36.79	0.109
	1100	36.25	36.18	0.193

## Data Availability

The original contributions presented in this study are included in the article. Further inquiries can be directed to the corresponding author.
